# Gait and plantar pressure changes after medial open-wedge high tibial osteotomy combined with arthroscopic partial meniscectomy

**DOI:** 10.3389/fbioe.2026.1760858

**Published:** 2026-08-12

**Authors:** Jizhe Lu, Zhiwei Peng, Qiang Gu, Xinming Wu, Zhexi Zhu, Guodong Liu, Hang Xu, Lei Tong

**Affiliations:** 1 The Second Affiliated Hospital of Xuzhou Medical University, Xuzhou, China; 2 Xuzhou Medical University, Xuzhou, China

**Keywords:** biomechanics, gait analysis, high tibial osteotomy, knee osteoarthritis, meniscectomy, plantar pressure

## Abstract

**Purpose:**

To evaluate the effects of medial open-wedge high tibial osteotomy (MOWHTO) combined with arthroscopic partial meniscectomy (APM) on spatiotemporal gait parameters and plantar pressure distribution in patients with varus knee osteoarthritis (KOA).

**Methods:**

A prospective study was conducted including 25 patients with medial compartment KOA who underwent MOWHTO combined with APM and 25 healthy controls. Gait parameters and plantar pressure were recorded preoperatively and 4 months postoperatively using the Zebris FDM pressure platform. Radiographic parameters—including the femorotibial angle (FTA), medial proximal tibial angle (MPTA), weight-bearing line ratio (WBLR), and tibial plafond inclination (TPI)—as well as clinical scores (VAS, HSS, WOMAC, and Lysholm) were assessed.

**Results:**

Postoperative radiographic parameters and clinical scores significantly improved (P < 0.001). Stride length (P < 0.05), gait velocity, and affected-limb stance duration (P < 0.01) increased. COP path length (P < 0.001) and gait temporal symmetry indices (P < 0.01) decreased. Radiographic correction correlated with plantar-pressure variables (P < 0.05). Medial forefoot (M1) and medial heel (HM) contact time and peak pressure also increased (P < 0.05).

**Conclusion:**

MOWHTO combined with APM was associated with improvements in lower-limb alignment, gait performance, plantar-pressure distribution, and gait symmetry during the short-term postoperative period.

## 1 Introduction

Knee osteoarthritis (KOA) is one of the most common degenerative joint diseases leading to chronic pain, impaired mobility, and reduced quality of life. Medial compartment degeneration is particularly frequent in patients with varus deformity (Dong et al., 2016; Haberkamp et al., 2020). For early to middle stages of medial KOA, medial open-wedge high tibial osteotomy (MOWHTO) is an effective joint-preserving procedure that corrects varus alignment by shifting the mechanical axis laterally, thereby redistributing the load from the medial to lateral compartment and reducing cartilage stress (Screpis et al., 2023).

However, many patients with medial compartment KOA present with concomitant meniscal degeneration or tears. While MOWHTO can correct lower-limb alignment and redistribute medial compartment loading, it does not directly address intra-articular pathology (Foreman et al., 2021; Zhan et al., 2023). Conversely, arthroscopic partial meniscectomy (APM) may relieve symptoms caused by unstable meniscal lesions but cannot correct malalignment (Kise et al., 2019). Therefore, combining MOWHTO with APM may influence postoperative biomechanics through both mechanical axis correction and improvement of meniscus-related symptoms, potentially affecting gait adaptation and plantar-pressure distribution (Lan et al., 2025; Park et al., 2023).

Previous studies after HTO have mainly focused on gait changes and radiographic outcomes, while plantar-pressure analysis has been less frequently investigated (Zhang et al., 2025). In addition, most biomechanical studies evaluated isolated osteotomy procedures, and evidence regarding plantar-pressure redistribution after MOWHTO combined with APM remains limited (Skvortsov et al., 2023). Postoperative changes in plantar-pressure distribution after HTO have been reported (Li et al., 2023); however, the correlations of bilateral gait symmetry and the degree of alignment correction with gait biomechanics have not been fully evaluated. Besides, patients with unilateral KOA often develop compensatory gait strategies characterized by asymmetric weight bearing and increased loading of the contralateral limb. Therefore, bilateral biomechanical assessment may provide further insight into postoperative functional recovery and load redistribution after MOWHTO combined with APM ((Liu et al., 2023), (Zeng et al., 2022)).

Therefore, this study aimed to quantitatively evaluate spatiotemporal gait parameters and plantar pressure distribution in both limbs of patients with medial KOA after MOWHTO combined with APM using a pressure-platform analysis system. We hypothesized that MOWHTO combined with APM would be associated with improved lower-limb alignment, more symmetrical gait characteristics, and redistribution of plantar pressure toward a more physiological loading pattern in both limbs.

## 2 Materials and methods

### 2.1 Study design and participants

This prospective study was approved by the institutional ethics committee (Approval No [2023]071,001). All participants gave written informed consent in accordance with the Declaration of Helsinki.

A total of 25 patients diagnosed with medial compartment knee osteoarthritis (KOA) who underwent medial open-wedge high tibial osteotomy (MOWHTO) combined with arthroscopic partial meniscectomy (APM) between July 2023 and March 2025 were included in the surgical group. An additional 25 age-, sex-, and body mass index (BMI)-matched healthy volunteers served as controls.

Inclusion criteria (Dong et al., 2016): age between 50 and 65 years, with body mass index (BMI)≤35 kg/m^2^ (Haberkamp et al., 2020); medial proximal tibial angle (MPTA) < 85° and varus deformity with mechanical axis deviation ≥ 5° (Screpis et al., 2023); radiographic evidence of medial compartment degeneration (Kellgren-Lawrence grade II-III) (Foreman et al., 2021); MRI-confirmed grade III medial meniscal injury accompanied by typical mechanical symptoms; and (Zhan et al., 2023) intact lateral compartment cartilage confirmed arthroscopically.

Exclusion criteria (Dong et al., 2016): History of trauma or surgery involving the knees or ankles (Haberkamp et al., 2020), any neurological or musculoskeletal condition that might affect gait or balance (e.g., cerebrovascular disease, lumbar spinal stenosis, flatfoot, pes cavus) (Screpis et al., 2023); severe osteoporosis or ligamentous instability; and (Foreman et al., 2021) fixed flexion deformity or inability to walk independently.

All surgical group participants underwent clinical, radiographic, and gait assessments preoperatively and 4 months postoperatively. The control group received a single randomized clinical and gait assessment.

### 2.2 Surgical procedure

All operations were performed by the same senior orthopedic team under general anesthesia. Standard diagnostic arthroscopy was first performed to evaluate intra-articular pathology. Arthroscopic partial meniscectomy (APM) was carried out to remove unstable or degenerative meniscal fragments and smooth the resection margins. Subsequently, a biplanar medial open-wedge high tibial osteotomy (MOWHTO) was performed through a 6–7 cm medial incision. The superficial medial collateral ligament was carefully released. Two parallel guide wires were inserted from the medial tibial cortex toward the tip of the fibular head, followed by osteotomy under fluoroscopic guidance. The osteotomy was gradually opened with a spreader to achieve the preoperatively planned correction angle. The gap was filled with an allograft bone block and stabilized using a TomoFix locking plate. After confirming satisfactory alignment, the wound was closed in layers. Postoperative radiographs were obtained to verify the correction angle and implant position.

### 2.3 Postoperative rehabilitation

Postoperative rehabilitation was conducted according to a standardized protocol supervised by the same physical therapy team. During the first postoperative week, quadriceps isometric exercises and ankle pump exercises were initiated. Continuous passive motion (CPM) training was introduced at postoperative weeks 2–4 and was gradually progressed to active range-of-motion exercises based on patient tolerance. Partial weight-bearing with crutch assistance was permitted at 3 weeks postoperatively, followed by progressive gait training and lower-extremity muscle strengthening exercises. After radiographic confirmation of preliminary osteotomy healing at postoperative weeks 4–6, patients were gradually transitioned to full weight-bearing ambulation without crutches under professional supervision. Daily activities were progressively resumed after 3 months postoperatively, with further advancement of muscle strengthening exercises. The detailed rehabilitation protocol is provided in the Supplementary Material–Supplementary Rehabilitation Protocol.

### 2.4 Radiographic evaluation

**FIGURE 1:**
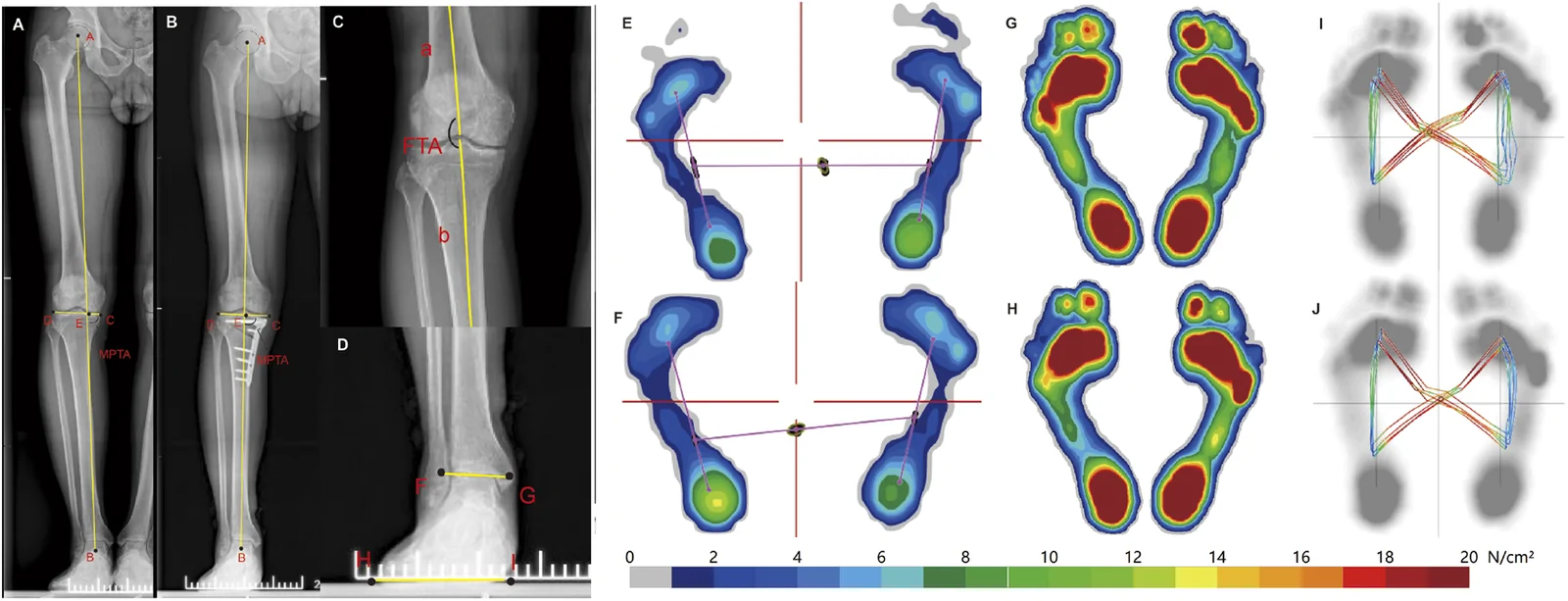
Radiographic, plantar-pressure, and gait evaluation of a representative patient pre- and postoperatively **(A,B)** Preoperative and postoperative full-length standing anteroposterior radiographs of the same patient. The medial proximal tibial angle (MPTA) is defined as the medial angle (∠CEB) formed between the tibial mechanical axis (line AB) and the tangent to the tibial plateau (line CD). The weight-bearing line ratio (WBLR) represents the percentage of the tibial plateau width traversed by the mechanical axis, calculated as CE/CD × 100%, where C is the intersection point of the mechanical axis with the tibial plateau and E is the medial edge of the plateau **(C)** The femorotibial angle (FTA) is the angle between the anatomical axis of the femur (line a) and that of the tibia (line b) **(D)** The tibial plafond–plantar angle (TPI) is the angle between the tangent to the distal tibial articular surface (line FG) and the plantar horizontal line (line HI) **(E,F)** Center of pressure (COP) alignment: the overall COP deviates from the midline preoperatively **(E)** and centralizes geometrically postoperatively **(F) (G,H)** Plantar-pressure thermogram: postoperatively **(H)**, maximum force increases in the left heel, with increased medial force concentration at the first metatarsal (M1) region compared to the preoperative **(G)** baseline **(I,J)** Gait butterfly parameters: the preoperative **(I)** left-shifted COP trajectory centralizes postoperatively **(J)**, demonstrating restored bilateral symmetry.

Radiographic evaluation was performed using full-length standing anteroposterior radiographs of both lower limbs, with the patella facing forward during imaging (Figures 1A–D). Radiographic measurements included (FTA)-the angle between the anatomical axes of the femur and tibia; MPTA-the medial angle between the tibial plateau and tibial shaft axis; WBLR-the percentage of the mechanical axis passing through the tibial plateau width; TPI-the angle between the tibial plafond and horizontal line. Measurements were performed independently by two blinded observers, and the average value was used for analysis. Interobserver reliability was tested in 10 randomly selected cases, showing excellent agreement (all ICCs >0.85).

### 2.5 Clinical evaluation

Subjective and functional outcomes were assessed using four validated clinical scoring systems: Visual Analog Scale (VAS) for pain intensity; Hospital for Special Surgery (HSS) knee score for joint stability and function; Western Ontario and McMaster Universities Osteoarthritis Index (WOMAC) for pain, stiffness, and physical function; Lysholm knee score for daily activity and sports performance.

### 2.6 Gait and plantar pressure analysis

**FIGURE 2:**
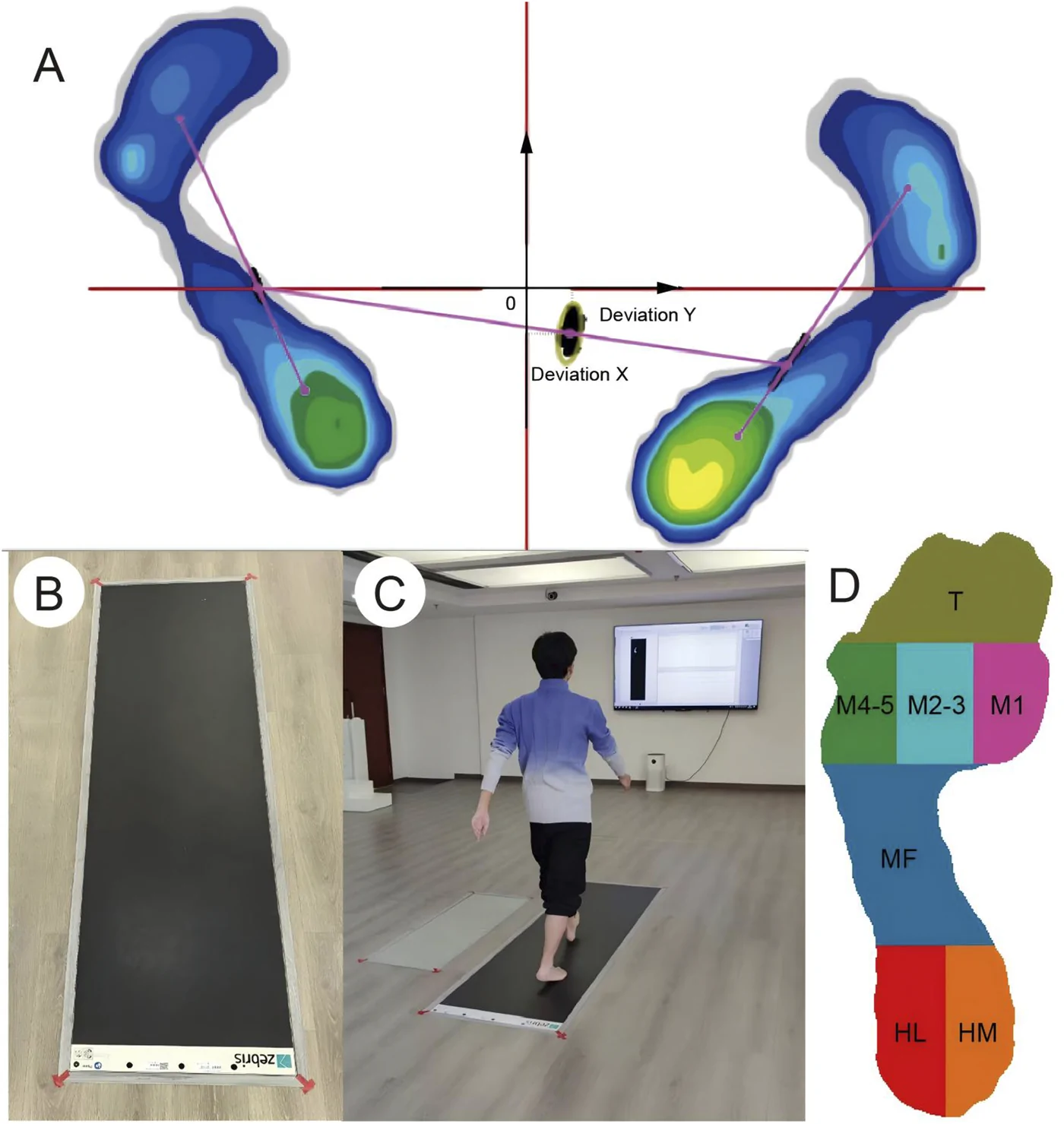
Spatiotemporal gait parameters and plantar pressure analysis **(A)** Static pedobarographic parameters, Deviation X and Y: the displacement of the center of pressure (COP) from the coordinate origin **(B)** A Zebris pressure distribution platform **(C)** A participant walking barefoot on the platform **(D)** The seven defined foot regions (T) toes (M1), medial forefoot (M2–3), central forefoot (M4–5), lateral forefoot (MF), midfoot (HM), medial heel (HL), lateral heel.

Dynamic plantar pressure and spatiotemporal gait parameters were measured using a pressure platform (FDM-2 system, Zebris Medical GmbH, Isny, Germany) (Figure 2B). The platform, equipped with 15,360 capacitive sensors, recorded data at a sampling frequency of 100 Hz and measured 212 cm × 60.6 cm in size (surface area = 1.285 m^2^). The reliability and repeatability of this system have been previously validated.

Static plantar biomechanics: For static testing, participants stood barefoot on the pressure platform with their feet shoulder-width apart and toes pointing forward. A 10-s segment of stable standing was selected for analysis (Figures 1E,F), generating a postural stability report that included the following parameters: center of pressure (COP) path length, mean velocity, COP area, and deviations along the X and Y-axes (Figure 2A).

Dynamic gait and plantar pressure assessment: Before testing, participants performed a 10-min warm-up to ensure a natural gait pattern and avoid intentional posture adjustments. Prior to data collection, the system was standardized according to each participant’s body weight. Participants were instructed to walk barefoot across a 6-m walkway containing the pressure platform at a self-selected comfortable speed. At the end of the walkway, they turned and walked back continuously until a 50-s recording was completed (Figure 2C).

During walking, dynamic positional and pressure data were collected by the platform’s sensor array and processed using Zebris FDM 1.18 software to generate a comprehensive gait analysis report (representative gait and plantar biomechanical curves are presented in Supplementary Figures S1–S3). The report included spatiotemporal gait parameters and plantar pressure variables (Plantar-pressure thermogram: Figures 1G,H; Gait butterfly parameters; Figures 1I,J). Spatiotemporal parameters included step length, step width, gait velocity, and foot progression angle (FPA), as well as the percentage of stance, swing, and double-support phases within a gait cycle. The foot progression angle was defined as the angle between the longitudinal axis of the foot and the direction of forward progression.

Regional plantar pressure analysis: Plantar pressure distribution was analyzed by dividing the footprint into seven anatomical subregions as defined by the Zebris software (Figure 2D). The software automatically recognized the foot outline and divided it into three regions--the forefoot, midfoot, and hindfoot--which were further subdivided as follows: Toe region (T). Metatarsophalangeal joint (MTPJ) region, divided into: MTPJ1 (medial forefoot, first metatarsal), MTPJ 2–3 (central forefoot, second and third metatarsals), MTPJ 4–5 (lateral forefoot, fourth and fifth metatarsals). Hindfoot region, divided vertically into: Medial heel (HM), Lateral heel (HL). For each region, contact time and peak force were recorded and normalized to body weight. The medial-to-lateral pressure ratio (MLPR) was calculated to evaluate medial-lateral load distribution during walking, defined as: Forefoot MLPR = MTPJ1/MTPJ4-5. Heel MLPR = HM/HL.

### 2.7 Statistical analysis

Sample size estimation was performed using G*Power 3.1. A preliminary test involving 14 participants (7 in the surgical group and seven in the control group) yielded an estimated effect size of Cohen’s f = 0.33 (medium effect) based on a mixed ANOVA (2 × 2 design). The required total sample size to achieve adequate power was 36 (18 per group). Considering potential dropout, the final sample included 25 patients in the surgical group and 25 matched healthy controls.

Statistical analysis was performed using IBM SPSS Statistics (IBM Corp., Armonk, NY, United States). The Shapiro-Wilk test was applied to assess data normality. Normally distributed variables were expressed as mean ± standard deviation (SD).

Paired-sample t-tests were used to compare preoperative and postoperative outcomes within the same subjects, including symmetry index (SI) parameters. Independent-sample t-tests were used for between-group comparisons. For non-normally distributed data, the Wilcoxon signed-rank test was applied.

For repeated measurements, mixed analysis of variance (mixed ANOVA) was performed to evaluate the interaction effects between time (preoperative vs. postoperative) and limb side (affected vs. unaffected). Bilateral limb data were analyzed within repeated-measures models, with limb side treated as a within-subject factor rather than as independent observations. When parametric assumptions were not met, the Brunner-Langer nonparametric mixed ANOVA was used.

Correlation analyses were performed to evaluate associations between radiographic correction parameters and changes in gait and plantar-pressure variables using Pearson correlation analysis. For variables not meeting normality assumptions, Spearman rank correlation analysis was applied.

Bonferroni-adjusted *post hoc* comparisons were applied where appropriate to reduce the risk of type I error caused by multiple testing. A two-tailed P value <0.05 was considered statistically significant.

## 3 Results

A total of 25 patients and 25 healthy controls were included in this study. There were no significant differences between the two groups regarding sex, age, BMI, or baseline clinical scores (P > 0.05). The mean follow-up duration was 4.23 months (Table 1).

**TABLE 1:** Demographic characteristics of participants.

Variable	Surgery group (n = 25)	Control group (n = 25)	P value
Sex (% female)	60%	52%	0.613
Age (years)	56.75 ± 11.85	57.25 ± 7.72	0.860
Weight (kg)	66.25 ± 11.3	63.2 ± 10.6	0.330
BMI (kg/m^2^)	25.16 ± 7.2	23.48 ± 5.4	0.355
Follow-up (months)	4.23 (3.8–5.1)	-	-

Postoperatively, lower-limb alignment parameters improved significantly (Table 2). The femorotibial angle (FTA), medial proximal tibial angle (MPTA), and weight-bearing line ratio (WBLR) all increased markedly (P < 0.001), whereas the tibial plafond-plantar angle (TPI) decreased significantly (P < 0.001), indicating effective correction of the mechanical axis.

**TABLE 2:** Radiographic alignment parameters before and after surgery.

Parameter	Preoperative	Postoperative	P value
FTA (°)	172.2 ± 3.9	181.3 ± 3.5	P < 0.001***
MPTA(°)	81.2 ± 2.3	89.2 ± 2.2	P < 0.001***
WBLR(%)	21.2 ± 16.4	58.6 ± 13.8	P < 0.001***
TPI(°)	5.8 ± 2.1	0.8 ± 1.5	P < 0.001***

FTA, femorotibial angle. MPTA, medial proximal tibial angle. WBLR, weight-bearing line ratio. TPI, tibial plafond–plantar angle. *Significance: ***P < 0.001.

Clinical outcomes also improved substantially (Table 3). Postoperative VAS and WOMAC scores decreased significantly, while HSS and Lysholm scores increased markedly (all P < 0.001). Postoperative VAS and WOMAC scores were comparable to those of the control group (P > 0.05), whereas HSS and Lysholm scores remained lower (P < 0.05), suggesting considerable improvement in pain and function though full recovery had not yet reached normal levels.

**TABLE 3:** Clinical functional scores.

Score	Preoperative	Postoperative	Control	P_1_ (Pre vs. Post)	P_2_ (Pre vs. Ctrl)	P_3_(Post vs. Ctrl)
VAS	5.12 ± 1.2	2.01 ± 1.1	1.4 ± 0.8	<0.001***	<0.001***	0.210
HSS	57.6 ± 2.4	83.2 ± 3.2	86.8 ± 1.2	<0.001***	<0.001***	0.012*
WOMAC	38.2 ± 3.4	17.8 ± 1.8	16.7 ± 1.7	<0.001***	<0.001***	0.310
Lysholm	52.8 ± 5.4	81.6 ± 3.5	84.2 ± 2.8	<0.001***	<0.001***	0.038*

VAS, Visual Analogue Scale. HSS, Hospital for Special Surgery Score. WOMAC, Western Ontario and McMaster Universities Osteoarthritis Index. Lysholm, Lysholm Knee Scoring Scale. *Significance: *P < 0.05, ***P < 0.001.

**FIGURE 3:**
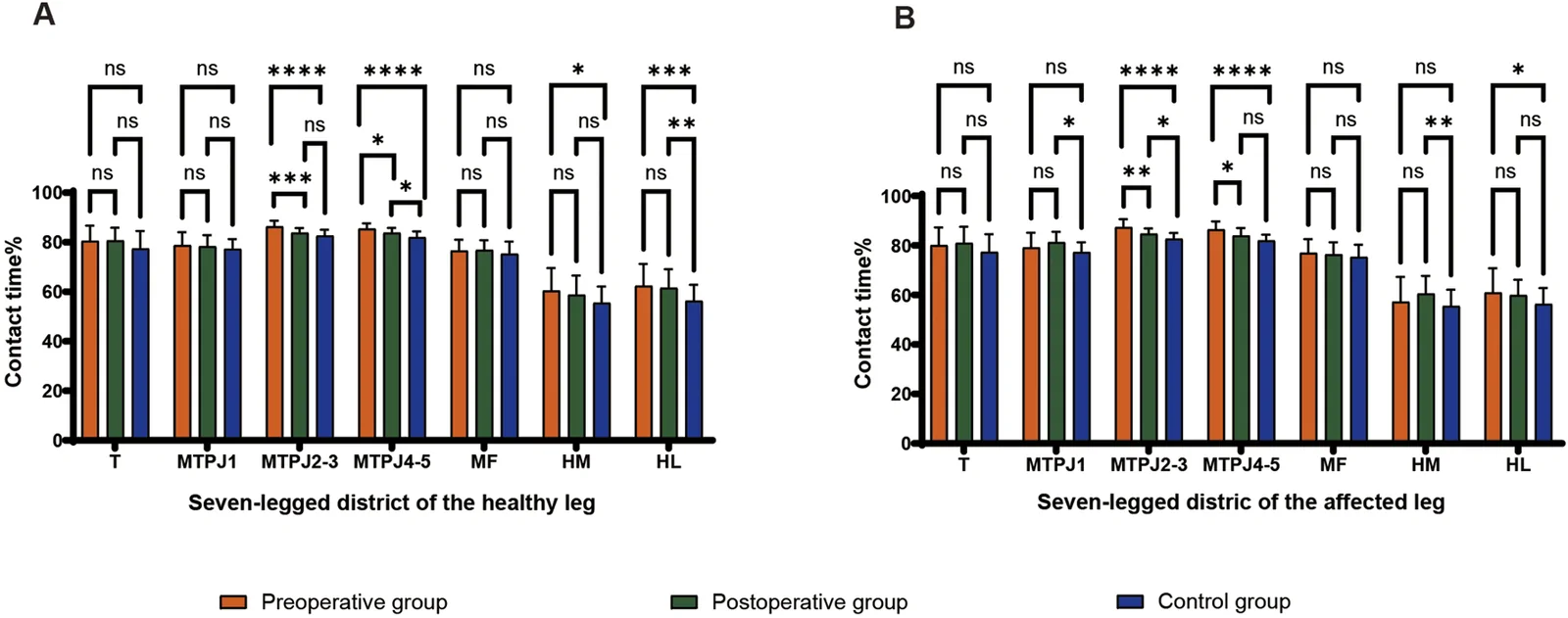
**(A)** Comparison of contact time across seven plantar regions of the unaffected limb among preoperative, postoperative, and control groups **(B)** Comparison of contact time across the same seven regions of the affected limb among the three groups. Data are expressed as mean ± standard deviation (SD). Statistical analysis was performed using mixed ANOVA (2 × 2 design). When assumptions for parametric testing were not met, the Brunner–Langer nonparametric mixed ANOVA was applied. Asterisks indicate statistically significant differences in contact time among groups for specific plantar regions, whereas N.S. denotes no significance. Significance levels: *P < 0.05; **P < 0.01; ***P < 0.001; ****P < 0.0001.N.S. = not significant.

**FIGURE 4:**
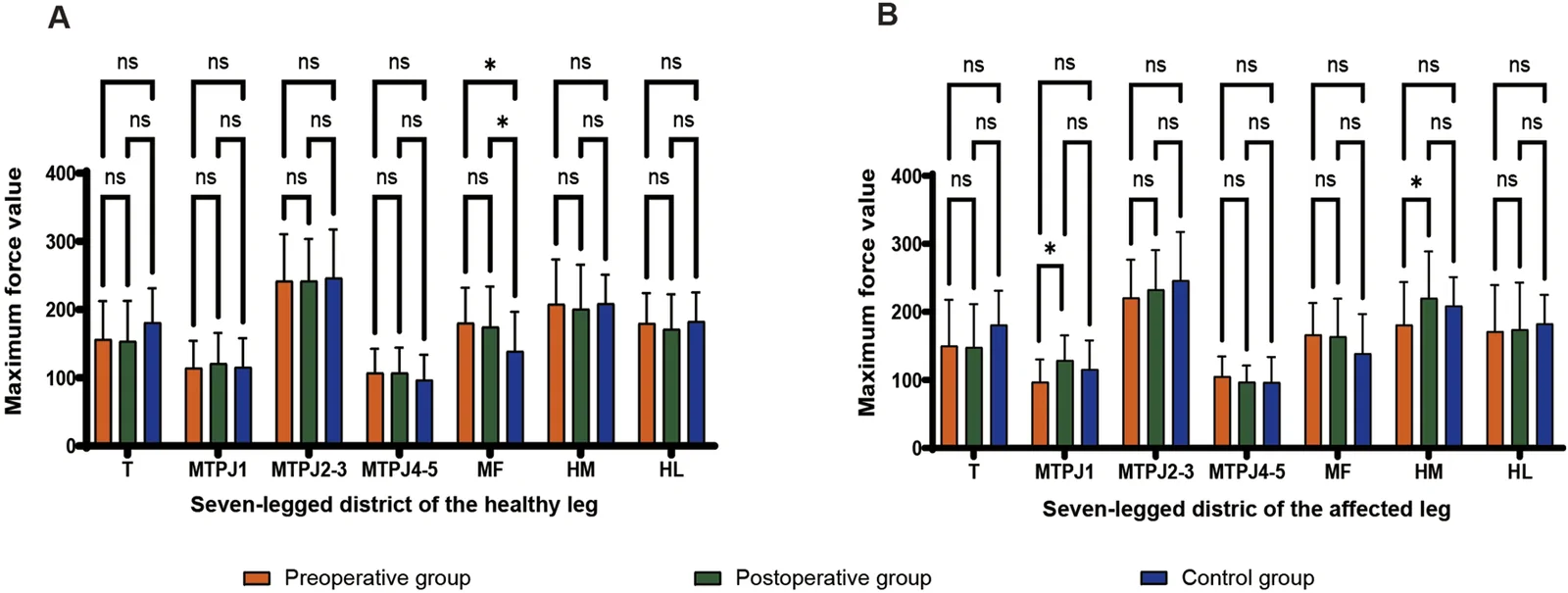
**(A)** Comparison of peak plantar pressure across seven regions of the unaffected limb among preoperative, postoperative, and control groups **(B)** Comparison of peak plantar pressure across the same seven regions of the affected limb among the three groups. Data are presented as mean ± standard deviation (SD). Statistical analysis was performed using mixed ANOVA (2 × 2 design). If parametric assumptions were violated, the Brunner–Langer nonparametric mixed ANOVA was used. Asterisks denote statistically significant differences in peak pressure among groups for specific plantar regions, whereas N.S. indicates no significance. Significance levels: *P < 0.05; **P < 0.01; ***P < 0.001; ****P < 0.0001.N.S. = not significant.

Gait parameters exhibited significant postoperative improvements (Table 4). Stride length and gait velocity increased significantly, while double-support time decreased (all P < 0.05). In the affected limb, stance phase duration increased and swing phase duration decreased (both P < 0.01), whereas step width and foot progression angle remained unchanged (P > 0.05). Significant postoperative changes in plantar-pressure distribution were observed (Figures 3, 4; Table 5). In the unaffected limb, the previously prolonged contact times in the forefoot (M2-3, M4-5) regions significantly decreased after surgery, with M2-3 and HM returning to normal levels. In the affected limb, peak pressure and contact time increased in the medial regions (M1 and HM) and decreased in the lateral regions (M4-5 and HL) (all P < 0.05). Heel MLPR increased significantly after surgery (P < 0.05), whereas forefoot MLPR did not differ from that of controls.

**TABLE 4:** Spatiotemporal gait parameters.

Variable	Preoperative	Postoperative	Control	P_1_ (Pre vs. Post)	P_2_ (Pre vs. Ctrl)	P_3_ (Post vs. Ctrl)
Step width (cm)	14.17 ± 3.29	13.89 ± 2.97	13.08 ± 2.51	0.520	0.190	0.290
Foot rotation angle (°, affected)	7.36 ± 4.40	7.23 ± 4.01	7.06 ± 6.17	0.860	0.780	0.880
Foot rotation angle (°, unaffected)	8.61 ± 5.50	8.54 ± 6.08	7.06 ± 6.17	0.940	0.270	0.310
Stride length (cm)	99.09 ± 22.06	104.6 ± 23.55	121.6 ± 12.76	0.040*	<0.001***	<0.001***
Step length (cm,affected)	49.22 ± 11.46	51.54 ± 11.47	61.92 ± 6.396	0.170	<0.001***	<0.001***
Step length (cm,unaffected)	49.87 ± 10.92	52.99 ± 12.06	61.92 ± 6.396	0.080	<0.001***	<0.001***
Walking speed (km/h)	3.02 ± 0.59	3.62 ± 0.67	4.16 ± 0.49	0.002**	0.001**	0.003**
Double support (%)	32.16 ± 5.61	29.08 ± 6.12	25.71 ± 2.27	0.006**	<0.001***	0.020*
Swing phase (%) affected	36.56 ± 1.87	34.84 ± 2.70	36.32 ± 1.45	0.005**	0.068	0.010*
Swing phase (%) unaffected	33.02 ± 2.7	34.10 ± 2.44	36.32 ± 1.45	0.092	<0.001***	0.004
Stance phase (%) affected	63.44 ± 1.87	65.16 ± 2.7	63.68 ± 1.45	0.005**	0.068	0.010*
Stance phase (%) unaffected	66.98 ± 2.7	65.90 ± 2.44	63.68 ± 1.45	0.092	<0.001***	0.004**

* Significance: *P < 0.05, **P < 0.01, ***P < 0.001.

**TABLE 5:** Center of pressure (COP) parameters and medial-lateral pressure ratios (MLPR).

Variable	Preoperative	Postoperative	Control	P_1_ (Pre vs. Post)	P_2_ (Pre vs. Ctrl)	P_3_(Post vs. Ctrl)
MLPR (forefoot)	0.96 ± 0.32	1.25 ± 0.32	1.33 ± 0.79	0.052	0.040*	0.068
MLPR (rearfoot)	1.07 ± 0.10	1.18 ± 0.20	1.16 ± 0.14	0.020*	0.040*	0.064
COP path length (mm)	109.64 ± 55.29	56.20 ± 37.51	39.70 ± 24.38	<0.001***	<0.001***	0.040*
COP mean velocity (mm/s)	11.28 ± 5.73	10.96 ± 5.34	10.32 ± 4.27	0.076	0.094	0.088
COP area (mm^2^)	234.64 ± 202.35	156.58 ± 118.94	129.75 ± 87.42	0.058	0.010*	0.062
COP deviation X (mm)	13.39 ± 11.16	6.31 ± 4.52	4.86 ± 2.52	<0.001***	0.002**	0.056
COP deviation Y (mm)	20.81 ± 12.56	11.43 ± 9.89	7.45 ± 5.76	0.001**	<0.001***	0.040*

MLPR, medial-to-lateral pressure ratio. COP, center of pressure. *Significance: *P < 0.05 ,**P < 0.01, ***P < 0.001.

The center of pressure (COP) path length decreased by approximately 49% (P < 0.001), and both COP area and lateral deviation comparable to control levels. Although longitudinal deviation improved, it remained slightly greater than in controls (P < 0.05) (Table 5).

**FIGURE 5:**
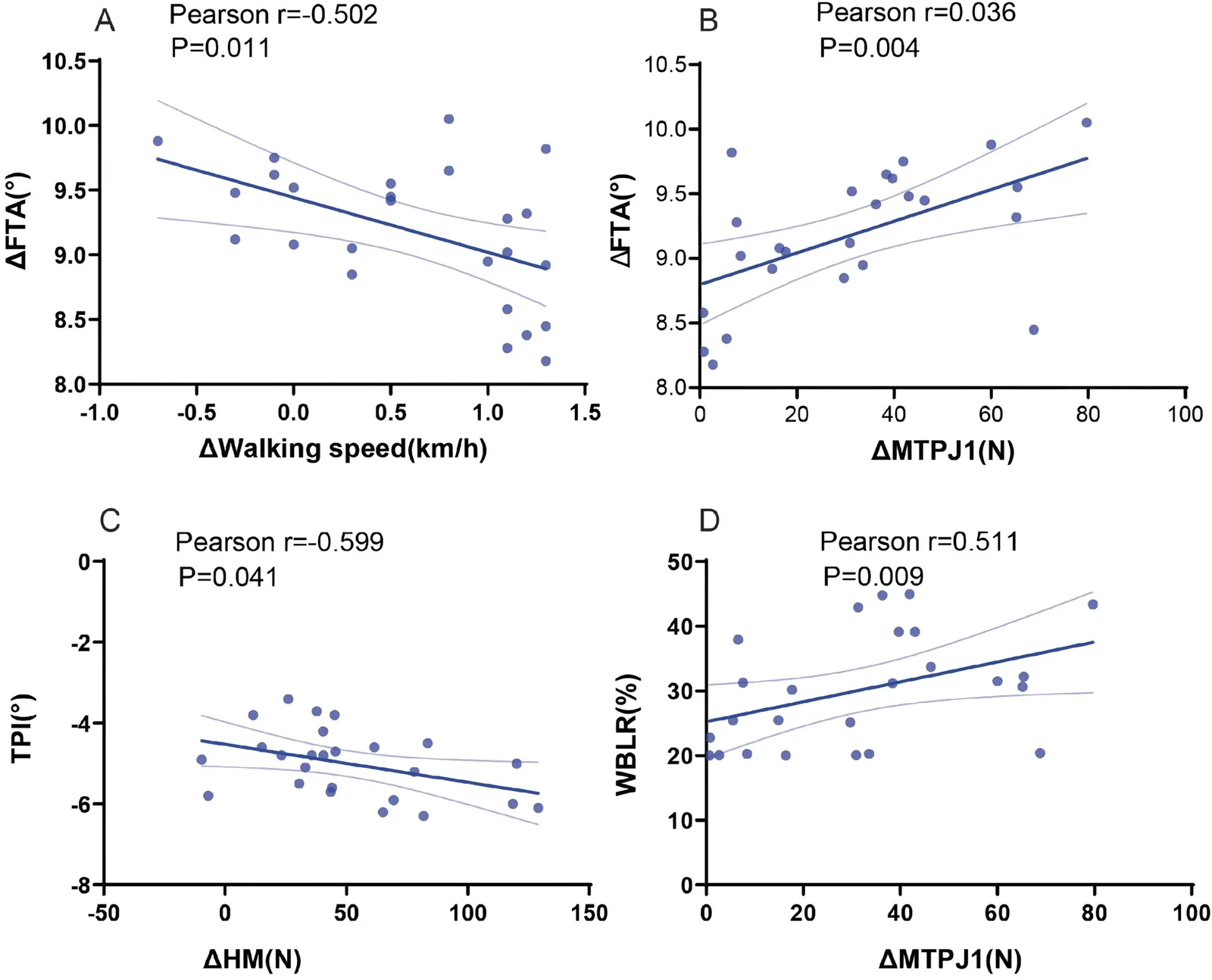
Correlation analyses between radiographic corrections and changes in gait and plantar-pressure parameters **(A)** Correlation between the change in femorotibial angle (ΔFTA) and the change in walking speed (ΔWalking speed) **(B)** Correlation between the change in femorotibial angle (ΔFTA) and the change in first metatarsophalangeal joint loading (ΔMTPJ1) **(C)** Correlation between the tibial plafond–plantar angle (TPI) and the change in heel medial loading (ΔHM) **(D)** Correlation between the change in weight-bearing line ratio (ΔWBLR) and the change in first metatarsophalangeal joint loading (ΔMTPJ1). The solid lines represent the linear regression lines, and the shaded areas indicate the 95% confidence intervals. Statistical analysis was performed using Pearson correlation analysis. If normality assumptions were violated, the Spearman rank correlation analysis was applied. The correlation coefficient (r) and P-values are displayed within each panel. Significance level: P < 0.05 indicates statistical significance.

Correlation analysis demonstrated that postoperative alignment correction was selectively associated with gait and plantar-pressure parameters (Table 6; Figure 5). Specifically, changes in FTA, TPI, and WBLR showed moderate correlations with rearfoot MLPR and MTPJ1 pressure (all P < 0.05). Furthermore, walking speed was moderately correlated with FTA, whereas HM pressure was moderately correlated with TPI (both P < 0.05). In contrast, changes in radiographic parameters were not significantly associated with double-support time, COP path length, or clinical outcome scores (VAS and WOMAC) (all P > 0.05).

**TABLE 6:** Correlations between changes in alignment parameters and gait parameters after surgery.

Variables	ΔFTA		ΔTPI		ΔWBLR	
	Pearson r	P value	Pearson r	P value	Pearson r	P value
ΔWalking speed (km/h)	−0.502	0.012*	0.228	0.273	−0.371	0.068
ΔDouble support (%)	−0.137	0.514	0.175	0.402	−0.194	0.352
ΔCOP path length (mm)	−0.321	0.117	0.351	0.085	−0.200	0.338
ΔMTPJ1(N)	0.552	0.004**	−0.412	0.041*	0.414	0.040*
ΔHM(N)	0.307	0.136	−0.411	0.041*	0.388	0.056
ΔMLPR (rearfoot)	0.490	0.013*	−0.599	0.002**	0.511	0.009**
ΔVAS	−0.321	0.118	0.293	0.155	−0.326	0.112
ΔWOMAC	−0.293	0.155	0.205	0.326	−0.138	0.512

Δvalue, the difference between postoperative and preoperative measurements. FTA, femorotibial angle. TPI, tibial plafond–plantar angle. WBLR, weight-bearing line ratio. COP, center of pressure. MTPJ1,medial forefoot, first metatarsal. HM, medial heel. MLPR, medial-to-lateral pressure ratio. VAS, Visual Analogue Scale. WOMAC, Western Ontario and McMaster Universities Osteoarthritis Index. *Significance: *P < 0.05, **P < 0.01.

**FIGURE 6:**
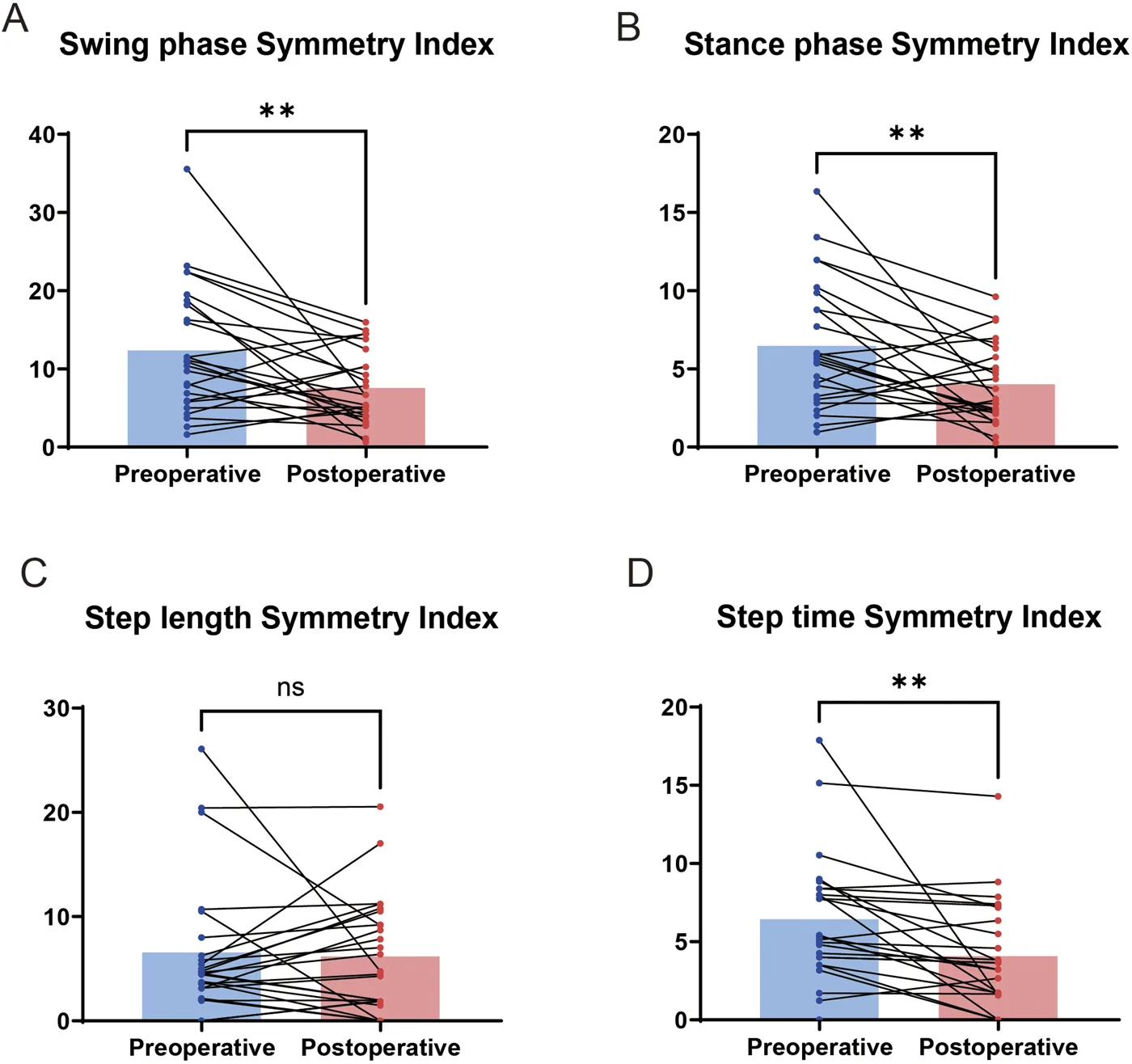
Preoperative and postoperative comparison of gait symmetry indices (SI) **(A–D)** Comparison of SI for swing phase **(A)**, stance phase **(B)**, step time **(C)**, and step length **(D)** within the same subjects. Data are presented as mean ± standard deviation (SD). Statistical analysis was performed using paired-sample t-tests. Significance levels: **P < 0.01.N.S. = not significant.

Symmetry index (SI) analysis demonstrated significant postoperative improvements in gait symmetry (Figure 6). Significant reductions were observed in swing phase, stance phase, and step time SI values following surgery (all P < 0.01), indicating improved bilateral gait balance. However, step length symmetry remained unchanged (P > 0.05).

Collectively, these findings demonstrate that MOWHTO combined with APM achieves radiographic alignment correction, which consequently triggers load redistribution, enhances gait symmetry, and promotes substantial clinical and functional recovery.

## 4 Discussion

The most important finding of this study is that MOWHTO combined with APM significantly improved lower limb alignment, gait symmetry, and plantar pressure distribution in patients with medial compartment knee osteoarthritis. Additionally, compensatory biomechanical alterations in the plantar pressure of the contralateral limb were observed, suggesting that rehabilitation strategies should also address the contralateral limb.

The present study confirmed that MOWHTO combined with APM significantly improved lower-limb alignment and clinical outcomes, consistent with previous reports (Lan et al., 2025; Vosoughi et al., 2025). Postoperatively, the WBLR reached 58.6%, which falls within the widely accepted target range of 55%–65%, indicating accurate correction of lower-limb alignment. In addition, the reduction in TPI further suggested alleviation of compensatory ankle varus alignment (Oh et al., 2023). We selected representative imaging parameters for correlation analysis and found significant associations between radiographic correction parameters and objective gait and biomechanical outcomes, whereas no significant correlations were observed with subjective pain or functional scores (VAS and WOMAC). This phenomenon may be explained by two factors. First, during the early postoperative period, biomechanical adaptation parameters may be more sensitive than patient-reported outcomes in reflecting the restoration of lower-limb alignment. Second, clinical scores are prone to a ceiling effect because of the substantial postoperative improvements, which may limit their ability to detect correlations (Clement et al., 2019). Meanwhile, although HSS and Lysholm scores improved markedly after surgery (Jacquet et al., 2021), they remained lower than those of healthy controls, indicating that functional recovery was still incomplete at the final follow-up. This finding suggests that recovery of overall knee function may lag behind anatomical correction and that further functional improvement may occur with longer-term recovery. Conventional radiographic and clinical evaluations may underestimate functional recovery. Integrating gait and plantar pressure analyses offers a more comprehensive, objective assessment of biomechanical restoration (Longo et al., 2025).

Systemic improvements in gait characteristics following HTO have been reported, including increased stride length and walking speed, prolonged single-limb stance, shortened double-support phase, and decreased knee adduction moment, all reflecting a redistribution of loads toward a more physiological pattern (Whatling et al., 2020). However, some studies have suggested that improvements in spatiotemporal gait parameters after HTO are limited, with primary benefits reflected in enhanced clinical scores (Jacquet et al., 2021). In the present study, postoperative stride length and gait velocity improved but remained below healthy control levels, indicating that gait recovery was incomplete at the final follow-up and may require a longer recovery period after alignment correction.

Preoperatively, due to pain and instability, patients often exhibited prolonged double-support phases as a compensatory strategy to enhance gait safety. Postoperatively, the shortened double-support time and restored stance-swing balance reflected improved gait coordination (Skvortsov et al., 2023; Valente et al., 2025). This was further supported by a statistically significant decrease in the symmetry indices (SI) for temporal parameters (swing phase, stance phase, and step time), indicating enhanced bilateral temporal symmetry. Moreover, the “antalgic gait” pattern observed preoperatively (Dong et al., 2025), characterized by weight transfer to the unaffected limb, was markedly alleviated postoperatively as pain decreased and alignment was corrected. With respect to foot progression angle, some studies have proposed that a moderate external foot rotation can reduce the second peak of the KAM(15). However, no significant changes were observed in the present study. This may be attributed to factors such as the tibial internal rotation induced by the osteotomy plane (Song and Kwon, 2022), or individual gait habits.

The center of pressure (COP) is a key biomechanical indicator for evaluating gait balance and postural control (Li et al., 2024). In the present study, COP path length was significantly reduced after surgery, while both mediolateral and anteroposterior deviations shifted toward the midpoint between the feet. Notably, the mediolateral deviation became comparable to the level of healthy controls, suggesting substantial improvement in balance control during standing and walking. Although COP area and mean velocity also showed decreasing trends postoperatively, these changes did not reach statistical significance, which may be attributable to the limited sample size and relatively short follow-up duration (Kurz et al., 2022). Furthermore, no significant correlations were identified between changes in COP path length and radiographic correction parameters. This finding suggests that the degree of alignment correction has little effect on the improvement of posture control. In addition, the relatively small sample size and the absence of subgroup analyses based on correction magnitude may have limited the ability to detect potential associations (Li et al., 2023). Overall, the observed improvements in COP parameters indicate enhanced load control and postural stability of the lower limbs following MOWHTO.

Plantar pressure analysis enables quantitative assessment of regional loading patterns during standing and walking, thereby indirectly reflecting changes in lower-limb alignment and compensatory mechanisms (Kamenaga et al., 2021). Previous studies found that patients with varus knee deformity often develop ankle varus and subtalar joint valgus compensations due to chronic medial compression, resulting in a lateralized plantar loading pattern (Diamond et al., 2024). By laterally shifting the mechanical axis, MOWHTO corrects varus deformity and significantly reduces TPI and talar varus angles (Van Oevelen et al., 2023), thereby altering the loading direction of the ankle-subtalar complex and redistributing plantar loads medially (Li et al., 2023). Correlation analysis demonstrated that a decrease in TPI was associated with increased peak pressures in the first metatarsophalangeal joint (MTPJ1) and medial heel (HM) regions, as well as an increase in rearfoot medial-lateral pressure ratio (MLPR), indicating a redistribution of plantar loads. Similar patterns were observed in the correlation analyses for other radiographic parameters. The present study confirmed this trend through seven-zone plantar pressure analysis: the medial-to-lateral pressure ratios in both forefoot and hindfoot regions increased significantly postoperatively, indicating an overall shift of plantar loads toward the medial side (De Pieri et al., 2023). Specifically, peak pressures in the medial heel (HM) and first metatarsal (M1) regions increased, with some values even slightly exceeding those of the control group. In theory, lateral pressures should correspondingly decrease; however, M4-5 and HL regions showed no marked reduction, suggesting that overall plantar loading increased after surgery. This phenomenon may be explained by the restoration of a natural “heel-strike to toe-off” propulsion pattern as pain subsides and gait characteristics improve (Wong et al., 2023). It is noteworthy that the compensatory adaptations developed preoperatively were not limited to the affected limb but also influenced the contralateral side through bilateral gait coupling, altering the overall lower-limb biomechanical pattern. Long-term compensatory loading may contribute to persistent alterations in plantar-pressure distribution on the contralateral side (Li et al., 2024). Although the mechanical axis was restored postoperatively, the peak pressure in the contralateral midfoot (MF) region remained elevated, suggesting that compensatory habits had not completely resolved. This contralateral adaptation reflects the “inertial effect” of biomechanical compensation and underscores the importance of bilateral lower-limb rehabilitation to prevent secondary overuse injuries on the contralateral side.

From the perspective of gait phases, the contact time of medial zones on the affected limb increased postoperatively, possibly reflecting mild compensatory varus alignment. Conversely, the contact time of the forefoot and heel on the contralateral limb decreased and became comparable to healthy control levels, indicating that the contralateral side gradually resumed its normal functional role rather than serving as the dominant weight-bearing limb. The rebalancing of bilateral loading may contribute to improved overall gait coordination (Wang et al., 2024).

This study has several limitations. First, the lack of an isolated MOWHTO control group prevents decoupling the specific contributions of APM from those of the osteotomy. Second, the 4-month follow-up was chosen because it represents a key milestone for returning to sports and active lifestyles. This timeframe is insufficient to capture long-term biomechanical and functional adaptations that may continue beyond this period. Third, the assessment relied solely on plantar pressure and gait parameters without joint-moment, surface electromyography, or 3D motion capture data, leaving the underlying mechanisms unverified. Fourth, the interrelationships among radiographic correction, clinical outcomes, and gait/plantar-pressure symmetry were not comprehensively explored. Finally, the relatively small sample size may limit statistical stability. Future long-term studies with larger, randomized cohorts and multimodal assessments are warranted to validate these findings.

## 5 Conclusion

Improvements in lower-limb alignment, clinical outcomes, gait characteristics, plantar-pressure distribution, and gait symmetry were observed following MOWHTO combined with APM, although some postoperative abnormalities persisted. Radiographic correction was associated with several biomechanical outcomes. Gait and plantar-pressure assessments may provide objective and quantitative information for evaluating postoperative recovery and guiding individualized rehabilitation.

## Data Availability

The raw data supporting the conclusions of this article will be made available by the authors, without undue reservation.
