# Supplementary material for: Gait and plantar pressure changes after medial open-wedge high tibial osteotomy combined with arthroscopic partial meniscectomy
Source: Front Bioeng Biotechnol. 2026 Aug 12;14:1760858. doi: 10.3389/fbioe.2026.1760858 (PMC13507774; doi:10.3389/fbioe.2026.1760858)

Supplementary Materials

1.Supplementary Rehabilitation Protocol

Postoperative Rehabilitation Protocol:A standardized, four-phase rehabilitation protocol was implemented for all patients.

Phase I (Week 1): Acute Protection.The primary goals were pain and edema control, thromboprophylaxis, and maintenance of quadriceps activation. Management included frequent active ankle pump exercises. Quadriceps isometric contractions and straight-leg raises were initiated on postoperative day 1. Patients were strictly maintained on non-weight-bearing (NWB) ambulation using crutches or a walker.
Phase II (Weeks 2 – 6): Early Rehabilitation and Partial Weight-Bearing.This phase focused on the gradual restoration of knee range of motion (ROM) and progressive muscle strengthening. Passive and assisted knee flexion exercises, supplemented by continuous passive motion (CPM) machines, were performed. Partial weight-bearing (PWB) was introduced between postoperative weeks 2 and 3, starting with toe-touch weight-bearing or 10% – 20% of body weight, and progressed based on clinical signs and radiographic evidence. The target ROM at the end of this phase (6 weeks) was set at ≥90° of knee flexion.
Phase III (Weeks 7 – 12): Functional Recovery and Full Weight-Bearing.Following radiographic confirmation of osseous healing (typically at 6 – 8 weeks), patients progressed to full weight-bearing status. Interventions expanded to include closed-chain strengthening, resistance training, functional step training, and progressive proprioceptive exercises. By 12 weeks, the targeted ROM was ≥120° of flexion with full extension.
Phase IV (Beyond 3 Months): Return-to-Activity.The focus shifted to the restoration of daily function and preparation for return to sports (RTS). Progressive lower-limb strengthening and endurance exercises were continued. Low-impact aerobic activities, such as cycling and swimming, were encouraged. Sport-specific training, including jogging and agility drills, was permitted after 6 months pending clinical clearance.
Follow-up and Adjunctive Measures.Patients were scheduled for clinical and radiographic evaluations at 1, 3, 6, and 12 months postoperatively to assess osteotomy healing and lower-limb alignment. Nutritional support, specifically adequate intake of protein, calcium, and vitamin D, was recommended to optimize bone healing. To prevent hardware complications or recurrence, high-impact sports and prolonged kneeling or squatting were discouraged throughout the first postoperative year.

2. Supplementary Legends

The following figure illustrates the preoperative plantar pressure curves of a single subject throughout the gait cycle.

2.1 Supplementary Figure S1

(Peak pressure in bilateral regions - N/cm²):this figure illustrates the changes in peak pressure across seven bilateral plantar regions during the gait cycle. Column A depicts the unaffected limb, while Column B illustrates the affected limb. The solid lines represent the mean pressure values, with the shaded areas indicating the standard deviation across the respective regions of interest.

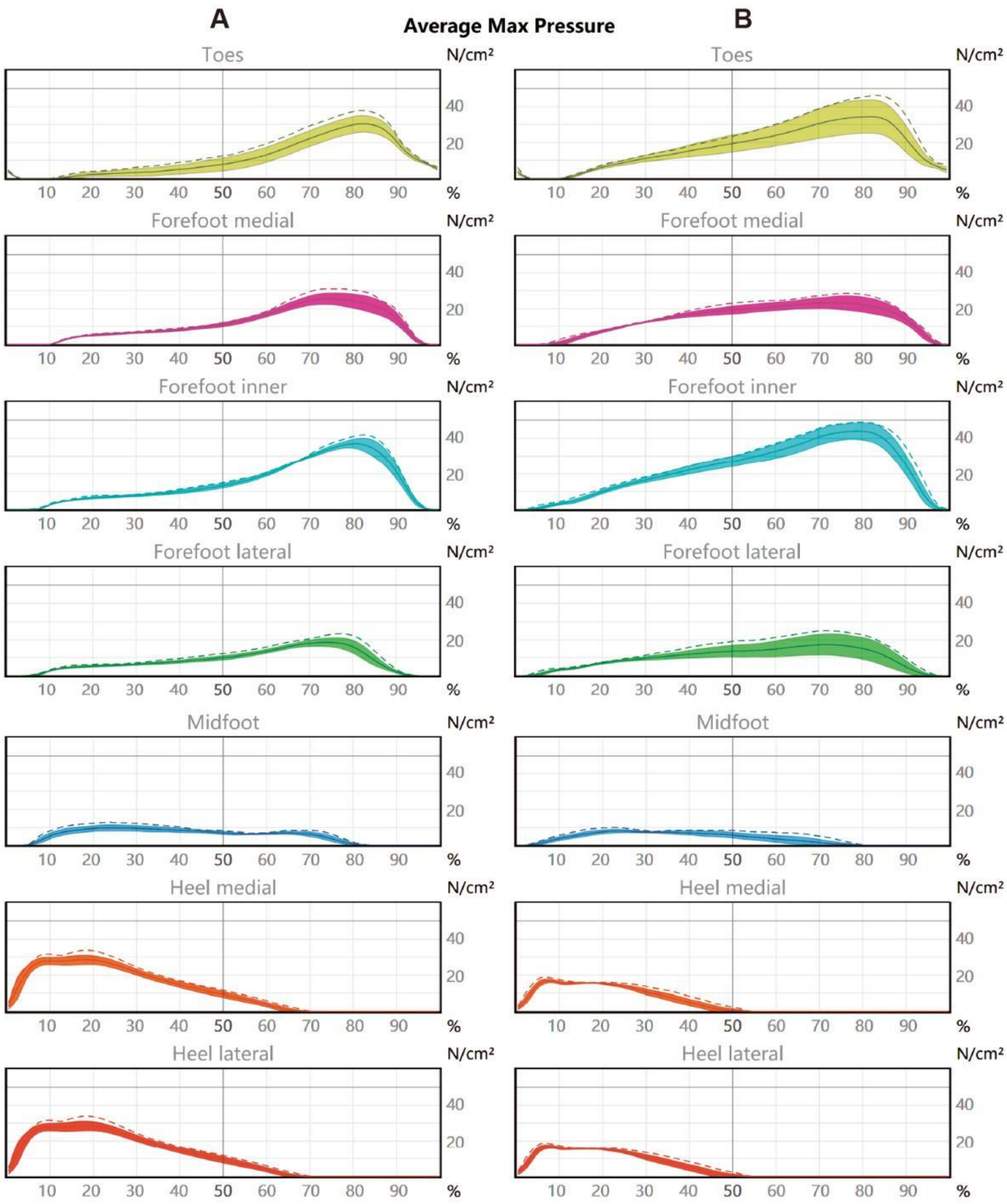

2.2 Supplementary Figure S2

(continuous pressure-time curve - N): this figure illustrates the changes in peak pressure across seven bilateral plantar regions during the gait cycle. Column A depicts the unaffected limb, while Column B illustrates the affected limb. The solid lines represent the peak pressure values, with the shaded areas indicating the standard deviation across the respective regions of interest.

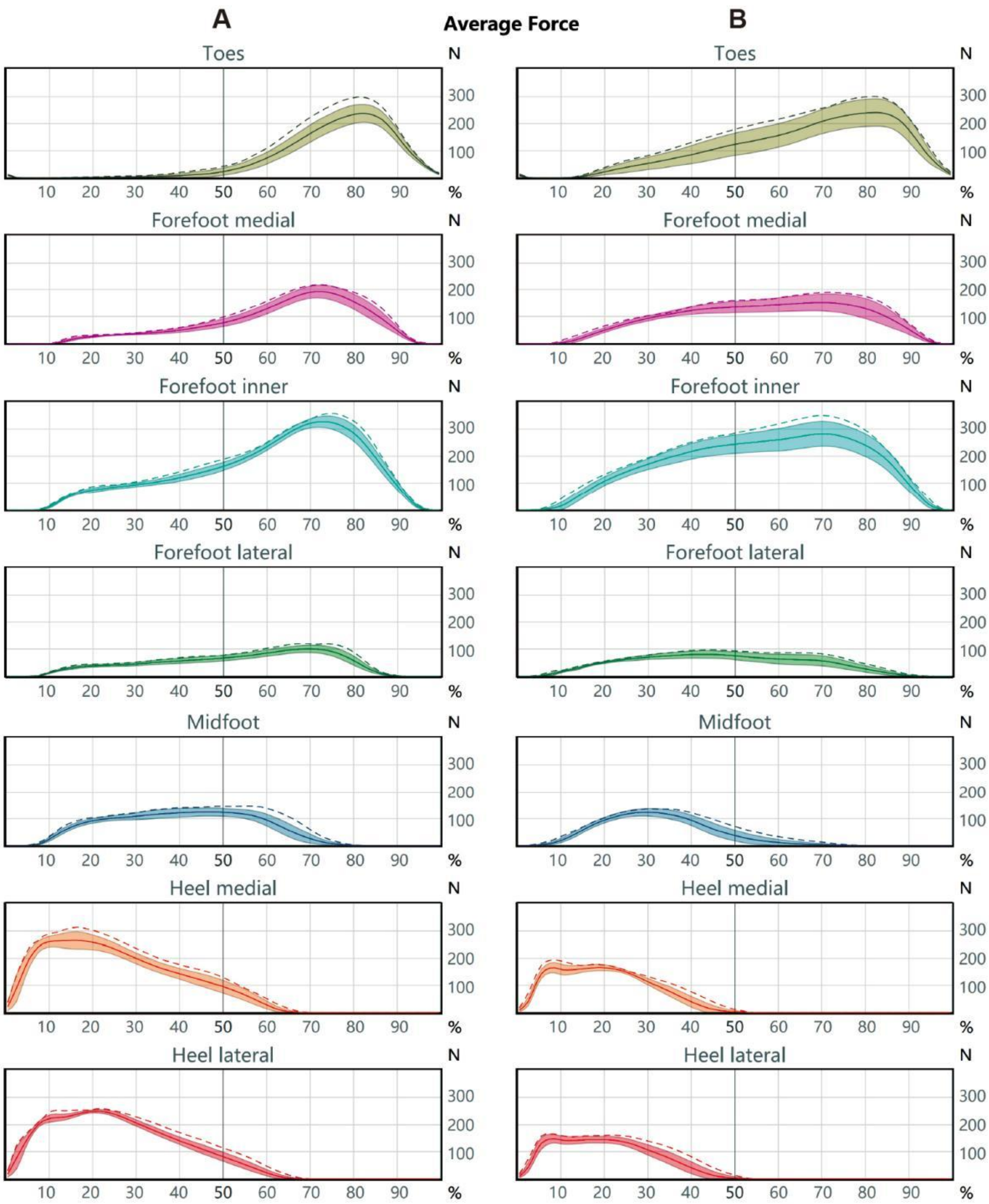

2.3 Supplementary Figure S3

(symmetrical loading pattern between forefoot and hindfoot): illustrates the sustained force distribution in the anterior (forefoot) and posterior (hindfoot) regions of both feet during static standing posture.

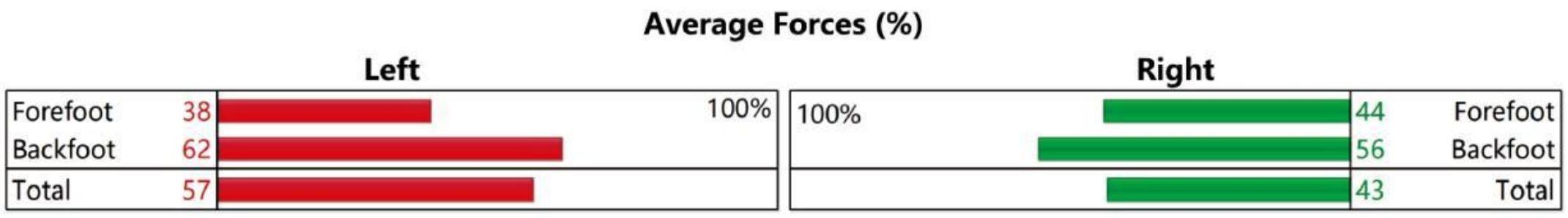

Supplement: Supplementary file 1 [file DataSheet1.pdf]
